# Synthesis and Characterization of Li_2_MgGeO_4_:Ho^3+^

**DOI:** 10.3390/ma15155263

**Published:** 2022-07-29

**Authors:** Nikola Bednarska-Adam, Marta Kuwik, Ewa Pietrasik, Wojciech A. Pisarski, Tomasz Goryczka, Bogusław Macalik, Joanna Pisarska

**Affiliations:** 1 Institute of Chemistry, University of Silesia, 9 Szkolna Street, 40-007 Katowice, Poland; marta.kuwik@us.edu.pl (M.K.); ewa.pietrasik@us.edu.pl (E.P.); wojciech.pisarski@us.edu.pl (W.A.P.); 2 Institute of Materials Engineering, University of Silesia, 75 Pułku Piechoty 1A Street, 41-500 Chorzów, Poland; tomasz.goryczka@us.edu.pl; 3 Institute of Low Temperature and Structure Research, Polish Academy of Sciences, Okólna 2 Street, 50-422 Wrocław, Poland; b.macalik@int.pan.wroc.pl

**Keywords:** Li_2_MgGeO_4_, Ho^3+^ ions, structure, luminescence

## Abstract

In this work, the synthesis and characterization of Li_2_MgGeO_4_:Ho^3+^ ceramics were reported. The X-ray diffraction measurements revealed that the studied ceramics belong to the monoclinic Li_2_MgGeO_4_. Luminescence properties were analyzed in the visible spectral range. Green and red emission bands correspondent to the ^5^F_4_,^5^S_2_→^5^I_8_ and ^5^F_5_→^5^I_8_ transitions of Ho^3+^ were observed, and their intensities were significantly dependent on activator concentration. Luminescence spectra were also measured under direct excitation of holmium ions or ceramic matrix. Holmium ions were inserted in crystal lattice Li_2_MgGeO_4_, giving broad blue emission and characteristic 4f-4f luminescent transitions of rare earths under the selective excitation of the ceramic matrix. The presence of the energy transfer process between the host lattice and Ho^3+^ ions was suggested.

## 1. Introduction

Holmium-doped materials are really interesting optical systems due to several 4f-4f electronic transitions operating in the visible and near-IR spectral ranges [1,2,3,4]. The energy level diagram of Ho^3+^ ions favors various radiative and non-radiative pathways dependent on the activator concentration, excitation wavelengths, host matrices, and their phonon energies. Trivalent holmium ions were introduced to numerous inorganic glasses [5,6,7,8,9], ceramics [10,11,12], and other nanoparticles synthesized using the sol–gel method [13]. Special attention has been paid to Ho^3+^-doped ceramic phosphors with superior luminescence properties. Ho^3+^-doped (K,Na)NbO_3_-based multifunctional ceramics present excellent optical temperature-sensing performance [14]. Such systems possess an optical temperature sensitivity higher than other rare-earth-doped ceramics or glasses, suggesting their attractive applications, especially in temperature-sensing devices. The same situation was also observed for similar Ho^3+^-doped ceramic compounds [15,16,17,18]. These aspects were also reported in the excellent review that was published recently [19]. Holmium-doped Y_2_Zr_2_O_7_ ceramics [20] were proposed as a new type of laser materials emitting visible light. The experimental results demonstrate that Y_2_Zr_2_O_7_:Ho^3+^ ceramics are able to generate efficient down- and upconversion luminescence. Further studies revealed that Y_2_O_3_-MgO:Ho^3+^ ceramics are novel IR-transparent nanocomposite ceramics [21] that can be applied to high-power eye-safe near-infrared lasers operating at about 2000 nm.

In this work, we show preliminary results for holmium-doped Li_2_MgGeO_4_:Ho^3+^ germanate ceramics. In particular, their visible emission properties under different excitation wavelengths were examined in detail. These germanate ceramic systems are less documented in the literature. The previously published works were mainly concentrated on luminescence investigations of Li_2_MgGeO_4_:Mn^2+^. Germanate ceramic Li_2_MgGeO_4_:Mn^2+^ was proposed as a green long-persistent phosphor [22]. Trap-controlled reproducible mechanoluminescent (ML) Li_2_MgGeO_4_:Mn^2+^ materials were also developed, and their short-term non-decaying ML behavior was reported [23]. Further investigations offering a constructive prospect for developing novel functional mechanoluminescent Li_2_MgGeO_4_:xMn^2+^ phosphors by defect control are also presented and discussed [24]. Optical properties of rare earths, especially Ho^3+^ ions in Li_2_MgGeO_4_ ceramics, have not yet been reported, to the best of our knowledge. Therefore, the synthesis and characterization of Li_2_MgGeO_4_:Ho^3+^ are exhibited here.

## 2. Experimental Methods

Germanate ceramics were characterized by a SETARAM Labsys thermal analyzer (SETERAM Instrumentation, Caluire, France) using the DSC method. The DSC curve was acquired in the range from room temperature to 1100 °C at the standard rate of 10 °C/min. The X-ray diffraction patterns were measured using an X’Pert-Pro diffractometer (PANalytical, Eindhoven, The Netherlands). The microstructure of germanate ceramics was observed using a JSM6480 scanning electron microscope (Jeol Ltd., Tokyo, Japan) as well as a JSM-7100F TTL LV (Jeol Ltd., Tokyo, Japan). The UV-VIS diffuse-reflectance spectra were collected using a Cary 5000 UV–VIS–NIR spectrophotometer, (Agilent Technology, Santa Clara, CA, USA). Excitation and luminescence measurements were carried out on a Photon Technology International (PTI) Quanta-Master 40 (QM40) UV/VIS Steady State Spectrofluorometer coupled with a tunable pulsed optical parametric oscillator (OPO) pumped by a third harmonic of a Nd:YAG laser (Opotek Opolette 355 LD, OPOTEK, Carlsband, CA, USA). The laser system included a double 200 mm monochromator, a xenon lamp as a light source, and a multimode UVVIS PMT (R928) (PTI Model 914, Horiba Instruments, New York, NY, USA) detector. The spectral resolution was equal to 0.5 nm. The Commission Internationale de I’Eclairage (CIE) chromaticity coordinates (x, y) and chromaticity diagram for Li_2_MgGeO_4_ ceramics doped with Ho^3+^ ions were calculated from the emission spectra and plotted using *Color Calculator* software (*Osram* Sylvania, Inc., Wilmington, MA, USA). Decay curves were recorded by a PTI ASOC-10 [USB-2500] oscilloscope (Horiba Instruments, New York, NY, USA) with an accuracy of ±0.5 µs. All experiments were carried out at room temperature.

## 3. Results and Discussion

### 3.1. Synthesis

Holmium-doped germanate ceramics with the following chemical compositions Li_2_Mg_(100−x)_GeO_4_:xHo^3+^ in molar % (where x = 0, 0.5, 2.5, and 5) were prepared by the solid-state reaction method using precursor powders of high purity (Sigma-Aldrich Chemical Co., St. Louis, MO, USA). The initial reagents Li_2_CO_3_ (99.997%), MgO (99.99%), GeO_2_ (99.99%), and optical active dopant Ho_2_O_3_ (99.999%) were weighed in stoichiometric amounts and mixed together homogeneously in an agate mortar for 1 h with ethanol (POCH Basic 96% pure) as a medium.

After the samples were grounded, the mixtures were calcinated in the muffle furnace (FCF 5 5SHP produced by Czylok, Jastrzębie-Zdrój, Poland) in a non-covered platinum crucible at 1100 °C for 6 h in the ambient air to achieve decarbonization. According to the results for similar Li_2_SrGeO_4_ phosphors obtained by Huang and Li [25], the following reaction takes place during calcination:

Li_2_CO_3_ + SrCO_3_ + GeO_2_ → Li^+^ + Sr^2+^ + Ge^4+^ + O^2−^ → Li_2_SrGeO_4_

Thus, it can be assumed that for the synthesized Li_2_MgGeO_4_:Ho^3+^ ceramics, referred to here as LMG-Ho^3+^, the following reaction occurs:

Li_2_CO_3_ + MgO + GeO_2_ + Ho_2_O_3_ → Li^+^ + Mg^2+^ + Ge^4+^ + O^2−^ + Ho^3+^ → Li_2_MgGeO_4_:Ho^3+^

The calcination process consisted of two steps. The temperature was increased to T = 800 °C in 30 min, then the furnace reached 1100 °C in 10 min. The samples were kept at this temperature for 5 h and 20 min.

In the next step, the granulated powders were divided into smaller batches and then mixed with an organic binder for 0.5 h. Among possible binders, polyvinyl alcohol (PVA) can be successfully used to receive germanate ceramic pellets. In general, PVA was often used as a binder [26,27,28,29]. The mixtures were uniaxially pressed into pellets of 10 mm diameter under 375 MPa. The prepared pellets were gradually heated to remove the PVA binder at 550 °C. The temperature was raised by 3 °C/min until the appropriate temperature was achieved. The samples were annealed for 2 h under ambient air conditions and naturally cooled to room temperature.

Lastly, the ceramic samples were sintered in a high-temperature furnace (FCF 4/170M produced by Czylok, Jastrzębie-Zdrój, Poland) at 1200 °C for 5 h. The thermal and structural results for olivine-type germanates presented previously and discussed by Koseva et al. [30] indicate that the annealing temperature of 1373 K (1100 °C) is optimal for obtaining well-crystallized phase-pure samples. It is worth noting that germanium dioxide has a low melting point at 1115 °C; therefore, the germanate olivines have low sintering temperatures [31]. In our synthesis procedure, the free sintering process consisted of several steps. First, the temperature was increased up to 800 °C for 1 h, and the samples were sintered without increasing the temperature for 15 min. Next, the furnace was heated to 1200 °C at a rate of 9 °C/min. The samples were sintered at the set temperature for 3 h. After this time, the resulting ceramics were cooled down to room temperature in a closed furnace.

### 3.2. Characterization

**Figure 1:**
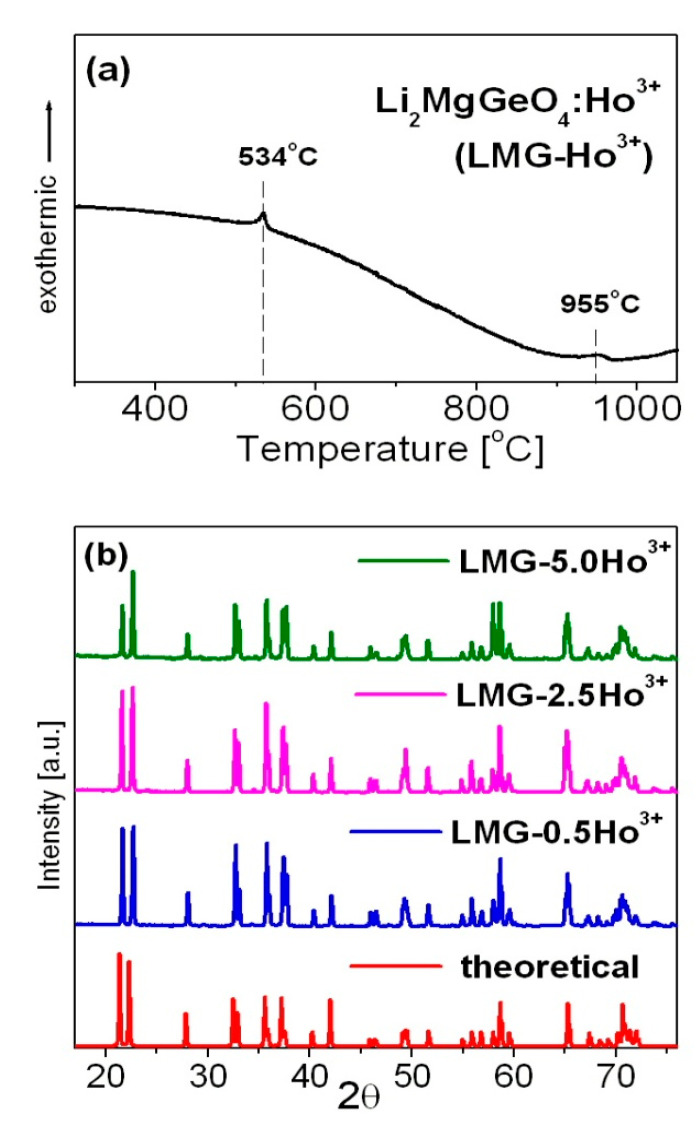
Representative DSC curve (**a**) and X-ray diffraction patterns (**b**) of LMG-Ho^3+^.

Figure 1 presents a representative DSC curve (a) and X-ray diffraction patterns (b) recorded for the studied LMG-Ho^3+^ ceramic system.

Li_2_MgGeO_4_:Ho^3+^ germanate ceramics (LMG-Ho^3+^) show two phase transitions [30]. The low-temperature phase transition near T = 534 °C is related to insignificant changes in the monoclinic symmetry. The high-temperature phase transition at about 955 °C is assigned to the transition from monoclinic to orthorhombic symmetry. The X-ray diffraction analysis confirmed that the studied LMG-Ho^3+^ ceramic systems crystallize in a monoclinic crystal lattice. The narrow diffraction lines are in a good agreement with the theoretical pattern belonging to the monoclinic Li_2_ZnGeO_4_ (ICDD PDF-4 database—card no 04-015-4929), which is isostructural to Li_2_MgGeO_4_ [32]. The schematic crystal structure of Li_2_ZnGeO_4_ belonging to the same monoclinic crystal system with P2_1_/n space group as Li_2_MgGeO_4_ was presented by Lin et al. [33].

**Figure 2:**
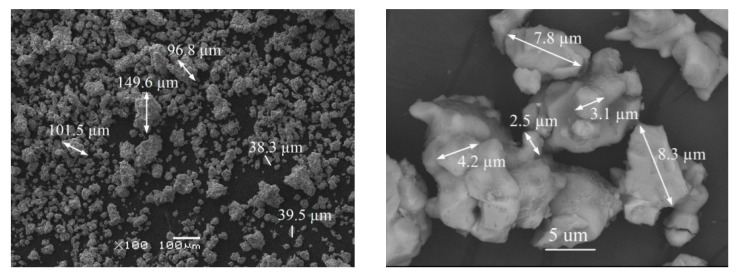
SEM images of LMG-Ho^3+^.

The introduction of Ho^3+^ playing the role of active doping ions to the host matrix Li_2_MgGeO_4_ do not cause any significant changes in the crystal structure. Independent of the Ho^3+^ concentration (0.5, 2.5, and 5 mol%), the XRD patterns without additional diffraction peaks due to impurities are almost consistent with pure Li_2_MgGeO_4_ monoclinic phase. This suggests that trivalent holmium ions are well-entered into the Li_2_MgGeO_4_ crystalline lattice. Furthermore, the Mg^2+^ ions are substituted by Ho^3+^ ions similar to Li_2_AGeO_4_ compounds, where the atomic positions of A^2+^ ions may be occupied by trivalent rare earth ions such as Ce^3+^, Tb^3+^, or Dy^3+^ [25]. The introduction of rare earth ions to the Li_2_MgGeO_4_ host matrix due to the substitution of a divalent cation (Mg^2+^) by a trivalent ion (Ho^3+^) can result in charge imbalance. It is well-known that charge compensation can be achieved by creating defects in the crystal lattice. The presence of defects in the ceramic host leads to the weakness of luminescent properties. However, the incorporation of alkali ions (Li^+^ or Na^+^) might compensate for the charge imbalance, reduce the lattice distortion, and enhance luminescence intensity without creating impurities in the host structure [34,35,36,37,38]. It should be also noted that agglomerates are present in the studied germanate ceramics. The diameter of agglomerates is changed from several dozen micrometers to even 150 µm. Most agglomerates have a diameter of around 40 µm. For the LMG-Ho^3+^ system, the grain sizes do not exceed 10 µm, which was verified using scanning electron microscopy (SEM). SEM images of the LMG-Ho^3+^ ceramic host are shown in Figure 2.

**Figure 3:**
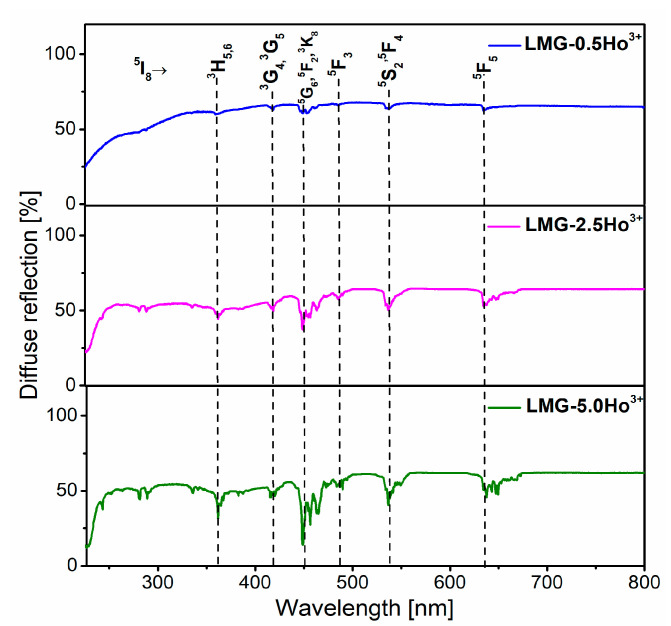
Reflectance spectra of LMG-0.5Ho^3+^, LMG-2.5Ho^3+^, and LMG-5.0Ho^3+^.

Figure 3 presents the UV-VIS diffuse-reflectance spectra for LMG-Ho^3+^ ceramics with different concentrations of holmium ions (0.5, 2.5, and 5 mol%) that were registered at room temperature in the 225–800 nm spectral region. Several narrow absorption peaks corresponding to intra-configurational 4f-4f electronic transitions from ground state ^5^I_8_ to higher-lying excited states of trivalent holmium are quite well observed. The registered absorption peaks are centered at about ~360 nm, ~415 nm, ~450 nm, ~535 nm, and ~635 nm and originated from the ^5^I_8_→^3^H_5,6_; ^5^I8→^3^G_4,5_; ^5^I_8_→^5^G_6_,^5^F_2_,^3^K_8_; ^5^I_8_→^5^F_3_; ^5^I_8_→^5^S_2_,^5^F_4_; and ^5^I_8_→^3^F_5_ transitions, respectively [39]. The strongest absorption, located at 450 nm, is attributed to the ^5^I_8_→^2^G_6_,^5^F_2_,^3^K_8_ transition of Ho^3+^ ions. The obtained results clearly show that with increasing optically active dopants the intensity of absorption peaks also increases, keeping the same intensity ratios between the recorded peaks.

**Figure 4:**
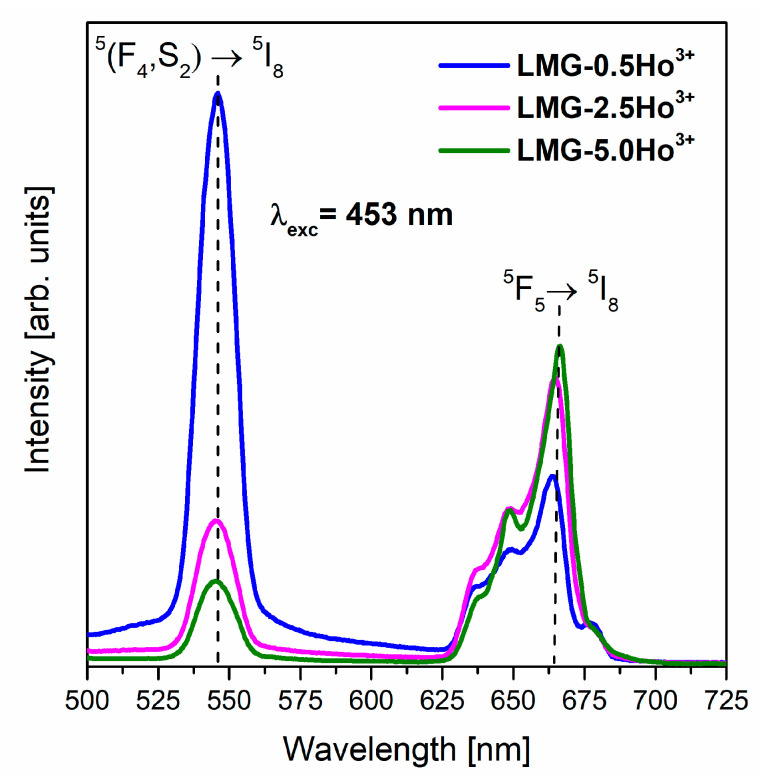
Luminescence spectra for LMG-Ho^3+^ ceramics with different contents of holmium ions.

The crucial aspect of analyzing the optical properties of transparent glass-ceramics, ceramics, and various phosphors is the characterization of the luminescence, which results from the 4f-4f electronic transitions of rare earth ions [40,41,42,43,44,45,46,47]. It is worth noting that studies of ceramic samples containing trivalent holmium ions are focused on characterizing the efficient green visible emission [6,18,48]. In general, as a result of the excitation of Ho^3+^ ions, the energy is transferred via non-radiative decay from higher-lying excited levels such as ^3^H_5_, ^3^H_6_, ^5^G_4_, ^5^G_5_, ^5^G_8_, ^5^F_2_, and ^5^F_3_ to levels ^5^F_4_, ^5^S_2_, and ^5^F_5_ of trivalent holmium ions. Afterward, the excitation energy is transferred to the ground state and causes emitting radiation due to characteristic transitions of Ho^3+^ ions. The emission spectra in the visible range, measured for the LMG-Ho^3+^ ceramic samples under the direct excitation of Ho^3+^ ions (λ_exc_ = 453 nm), consist of two well-separated bands (Figure 4).

Independent of the concentration of holmium ions in LMG ceramics, the emission bands located at 546 nm and 666 nm, corresponding to electronic transitions originating from the ^5^F_4_, ^5^S_2_, and ^5^F_5_ states to the ground state, ^5^I_8_, were recorded, respectively. Both the band originating from the ^5^F_4_,^5^S_2_→^5^I_8_ transitions and the band associated with the ^5^F_5_→^5^I_8_ transition of Ho^3+^ ions are characterized by considerable emission intensity.

Our investigations clearly indicate that the relative integrated emission intensities due to the ^5^F_4_,^5^S_2_→^5^I_8_ (green) and ^5^F_5_→^5^I_8_ (red) transitions of Ho^3+^ ions in Li_2_MgGeO_4_ ceramics depend critically on activator concentration. The emission intensity of the green transition decreases, whereas the emission intensity of red transition is enhanced, with increasing Ho^3+^ ion concentrations. The observed intensities of green (546 nm) and red (666 nm) emissions for our ceramic samples are opposite in comparison to the CaLa_4_-xSi_3_O_13_:Ho^3+^ phosphors reported by Singh et al. [49]. In this study, the CaLa_4_-xSi_3_O_13_ ceramics doped with holmium ions exhibited an intense green emission at 546 nm attributed to the ^5^F_4_,^5^S_2_→^5^I_8_ transitions, whereas the red emission from the ^5^F_5_→^5^I_8_ transition was significantly weaker. Moreover, Li et al. [50] registered dominant green emission under the direct excitation of Ho^3+^ ions in potassium sodium niobate-based ceramics. However, the emission intensity of the band corresponding to the ^5^F_4_,^5^S_2_→^5^I_8_ transitions decreased with increasing contents of optical active dopants (Ho^3+^) in ceramic compositions. This phenomenon is attributed to the so-called the concentration luminescence quenching effect, which was also observed for holmium-doped LiPbB_5_O_9_ [51]. The results obtained for LiPbB_5_O_9_:Ho^3+^ indicated that the intensity of visible emission was increased for samples with low contents of Ho^3+^ (from 0.04 mol% to 0.08 mol%) and then decreased with further increases in the holmium ion concentration. Consequently, once the content of optical active dopants exceeds a critical value, the mechanism of concentration quenching is triggered [51]. The origin of this phenomenon is due to non-radiative energy transfer interactions between emitting activator ions. Energy transfer between them is more effective at high concentrations because the average distance between the activator ions is shorter, and the energy transfer probability is higher. Thus, the coupled excited states ^5^F_4_ and ^5^S_2_ (Ho^3+^) are quite easy depopulated, and green emission is effectively quenched in the case of highly holmium-concentrated systems. These effects are due to cross-relaxation channels, i.e., the resonant cross relaxation processes (^5^S_2_ + ^5^F_4_:^5^I_8_)→(^5^I_4_:^5^I_7_) and (^5^S_2_ + ^5^F_4_:^5^I_8_)→(^5^I_7_:^5^I_4_) as well as the phonon-assisted cross-relaxation (^5^S_2_ + ^5^F_4_:^5^I_8_)→(^5^I_6_:^5^I_7_). It was presented well and discussed earlier for LiPbB_5_O_9_:Ho^3+^ [51].

Additionally, the CIE chromaticity coordinates (x, y) for the studied LMG ceramic samples excited at 453 nm were calculated from the registered emission spectra in the visible range. The results are given in Table 1. It is clearly seen that the increasing concentrations of holmium ions in Li_2_MgGeO_4_ germanate ceramics caused changes in the x and y coordinates of the chromaticity parameters.

**Figure 5:**
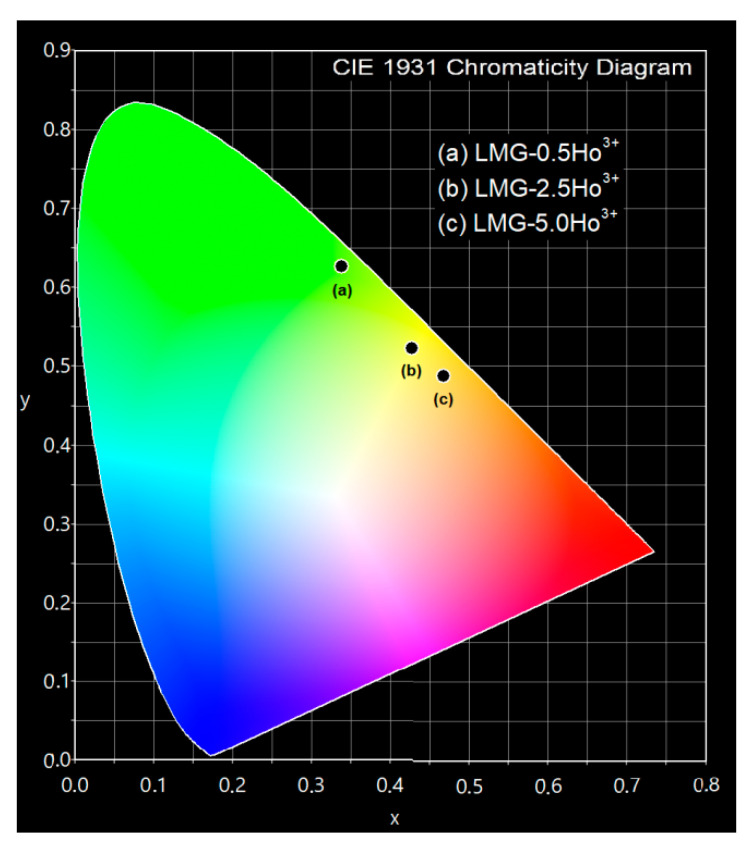
The influence of Ho^3+^ concentration on chromaticity coordinates for Li_2_MgGeO_4_ ceramics.

The chromaticity diagram shows the luminescence color modification from green through yellowish to nearly orange for ceramic systems doped with 0.5, 2.5, and 5 mol% Ho^3+^ concentrations, respectively. It is illustrated in Figure 5.

**Figure 6:**
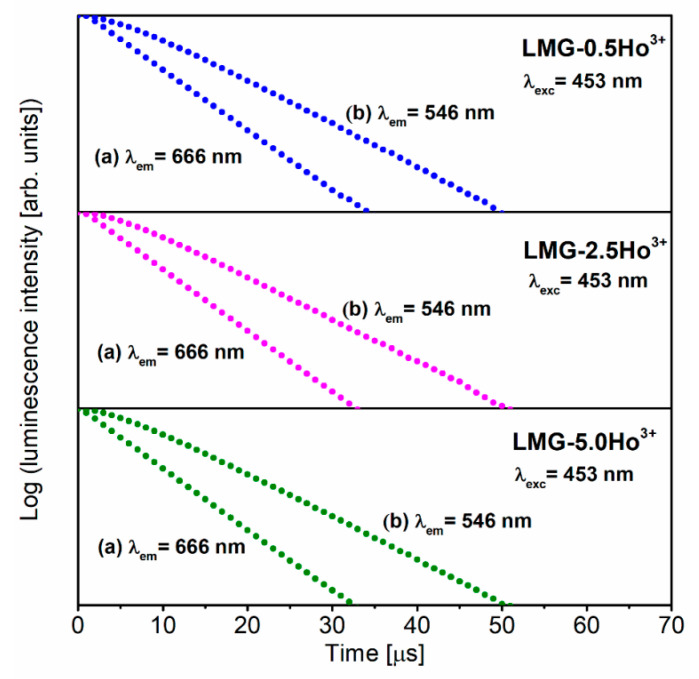
Decay curves for LMG-Ho^3+^ ceramics with different contents of holmium ions.

Luminescence decays from the excited states of holmium ions in Li_2_MgGeO_4_ ceramics were carried out to fully characterize the studied systems. It is clearly seen in Figure 6 that all registered decay profiles are nearly exponential, independent of the content of optically active dopants. Based on the decays, the luminescence lifetimes for the ^5^F_4_ + ^5^S_2_ (λ_em_ = 546 nm) and ^5^F_5_ (λ_em_ = 666 nm) levels of Ho^3+^ were evaluated. The τ_m_ values for LMG-Ho^3+^ ceramics are nearly 10 μs (^5^F_4_ + ^5^S_2_) and 6 μs (^5^F_5_). Moreover, it was observed that this spectroscopic parameter is practically independent of the concentration of Ho^3+^ ions in ceramic samples. The measured emission lifetimes for the ^5^F_4_ + ^5^S_2_ states of Ho^3+^ in the LMG host are similar to fluorophosphate glass [6], but they are relatively lower compared to other reported glasses [7], ceramics [52], and crystals [53].

### 3.3. Luminescence Properties under Different Excitation Wavelengths

**Figure 7:**
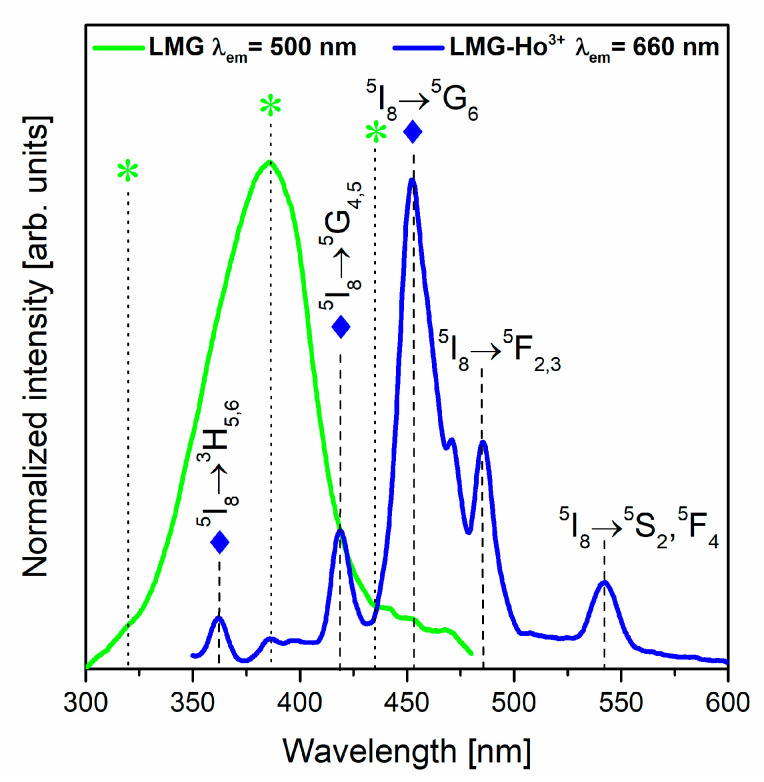
Excitation spectra of LMG and LMG-Ho^3+^ ceramics.

In order to study the luminescence properties of LMG-Ho^3+^ ceramics, the excitation spectra were registered at selected monitoring wavelengths. The results are presented in Figure 7. The excitation spectrum for LMG monitored at λ_em_ = 500 nm shows a broad band located at about 385 nm. Based on previous investigations, it was found that this phenomenon is related to the occurrence of magnesium in ceramics. It was proposed that the band in this spectral region is assigned to the excitation band of MgO [54,55,56,57]. On the other hand, the excitation spectrum for the LMG-Ho^3+^ ceramic monitored at the emission wavelength λ_em_ = 660 nm consists of several narrow and well-resolved bands. These bands were assigned to characteristic electronic transitions originating from the ground state, ^5^I_8_, to the higher-lying states of trivalent Ho^3+^. The following transitions of Ho^3+^ ions centered at about 453 nm, 485 nm, and 543 nm were identified as ^5^I_8_→^5^G_6_, ^5^I_8_→^5^F_2,3_, and ^5^I_8_→^5^S_2_,^5^F_4_, respectively. Among the registered bands, the most intense band is located near 453 nm, corresponding to the ^5^I_8_→^5^G_6_ (λ_em_ = 453 nm) transition of Ho^3+^. Moreover, it was also observed in the 350–430 nm spectral region that bands assigned to the ^5^I_8_→^3^H_5,6_ and ^5^I_8_→^5^G_4,5_ transitions of Ho^3+^ overlapped with the excitation of the ceramic matrix. Therefore, it is possible to obtain the characteristic luminescence associated with trivalent Ho^3+^ ions by the direct excitation of the LMG host. This hypothesis was verified using the emission spectra measurements at selected excitation wavelengths. The ceramic samples were excited at the appropriate wavelengths assigned directly to the transitions of Ho^3+^ as well as the LMG ceramic matrix, and they are referred to by diamonds and asterisks in Figure 7.

**Figure 8:**
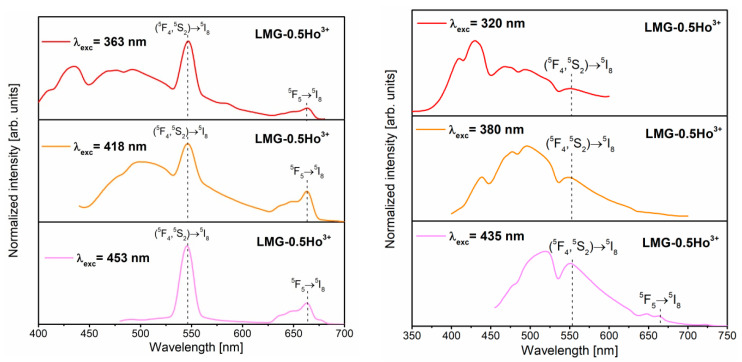
Emission spectra of LMG-Ho^3+^ under direct excitation of holmium ions (**left**) and host matrix (**right**).

Figure 8 presents the emission spectra of LMG-0.5Ho^3+^ ceramics measured under the direct excitation of holmium ions (on the left) and the ceramic matrix (on the right). In the spectra, a broad luminescence band with a tail extending to about 700 nm could be distinguished as originating from the ceramic matrix. It is also worth emphasizing that the broad band from the LMG ceramic matrix overlapped with the narrow emission bands characteristic of Ho^3+^ ions. Depending on the excitation wavelengths, blue or greenish-blue broad emission was observed, similar to the excellent results obtained previously for MgO films, and these phenomena are related to the occurrence of defects and oxygen vacancies in MgO assigned to F-type centers [57]. After analyzing the literature data, it is suggested that the broad blue emission band is due to the presence of magnesium in the LMG ceramic matrix.

The spectroscopic analysis indicates that the relative intensities of the emission bands attributed to the LMG host and Ho^3+^ ions are significantly dependent on the excitation wavelength. The green and red emission bands associated with ^5^F_4_,^5^S_2_→^5^I_8_ and ^5^F_5_→^5^I_8_ transitions are the most intense for ceramic samples during the direct excitation of Ho^3+^ ions. Additionally, the emission spectra of LMG-0.5Ho^3+^ samples excited at 363 nm and 418 nm contain broad blue band characteristic for the ceramic matrix. This is due to the fact that these excitation lines of Ho^3+^ overlap with the excitation spectrum of the matrix, as mentioned above. The characteristic band corresponding to the emission of the ceramic matrix becomes insignificant in the spectrum recorded upon the excitation by the 453 nm line.

Interesting results were obtained for LMG-0.5Ho^3+^ samples under the direct excitation of the ceramic matrix. The samples were excited at 320, 380, or 435 nm. The luminescence intensities of the broad band characteristic for the ceramic matrix were considerably higher in reference to the results obtained for the same LMG-0.5Ho^3+^ samples but measured under the direct excitation of holmium. Additionally, green emission bands at 550 nm, due to the ^5^F_4_,^5^S_2_→^5^I_8_ transitions of Ho^3+^, are also well-observed during the excitation of the ceramic matrix. The presence of characteristic bands for trivalent holmium ions may indicate the energy transfer between the LMG ceramic matrix and the optical active dopants. Further investigations are in progress.

## 4. Conclusions

In this work, preliminary results for Li_2_MgGeO_4_:Ho^3+^ ceramics, referred to as LMG-Ho^3+^, are presented and discussed. The X-ray diffraction analysis confirmed the presence of crystalline phase belonging to the monoclinic Li_2_MgGeO_4_. The luminescence spectra consisted of characteristic narrow bands in the green and red spectral regions, which correspond to the ^5^F_4_,^5^S_2_→^5^I_8_ and ^5^F_5_→^5^I_8_ transitions of Ho^3+^ ions. Their relative integrated emission intensities depend significantly on activator concentration. The emission intensity of the green band decreased rapidly, whereas the intensity of the red emission was enhanced by increasing Ho^3+^ concentrations. In particular, the emission spectra of Li_2_MgGeO_4_:Ho^3+^ were examined under different excitation wavelengths. The emission spectra were measured under the direct excitation of Ho^3+^ ions and the ceramic matrix. The spectroscopic analysis revealed the presence of broad blue luminescence, which was assigned to the ceramic matrix, and narrow emission bands characteristic of the 4f-4f transitions of Ho^3+^. Further studies suggest an energy transfer between the LMG ceramic matrix and the Ho^3+^ ions.

## Figures and Tables

**Table 1:** The values of CIE chromaticity coordinates for LMG-Ho^3+^ ceramic systems.

Ceramic Code	CIE Coordinates	
	x	y
LMG-0.5Ho^3+^ LMG-2.5Ho^3+^ LMG-5.0Ho^3+^	0.338 0.427 0.468	0.627 0.523 0.488

## Data Availability

Not applicable.
